# Fibrinogen Activates the Capture of Human Plasminogen by Staphylococcal Fibronectin-Binding Proteins

**DOI:** 10.1128/mBio.01067-17

**Published:** 2017-09-05

**Authors:** Philippe Herman-Bausier, Giampiero Pietrocola, Timothy J. Foster, Pietro Speziale, Yves F. Dufrêne

**Affiliations:** a Institute of Life Sciences, Université catholique de Louvain, Louvain-la-Neuve, Belgium; b Department of Molecular Medicine, Unit of Biochemistry, University of Pavia, Pavia, Italy; c Department of Microbiology, Trinity College Dublin, Dublin, Ireland; d Walloon Excellence in Life Sciences and Biotechnology (WELBIO), Wallonia, Belgium; University of Washington

**Keywords:** cell wall, ligand binding, *Staphylococcus aureus*, surface proteins

## Abstract

Invasive bacterial pathogens can capture host plasminogen (Plg) and allow it to form plasmin. This process is of medical importance as surface-bound plasmin promotes bacterial spread by cleaving tissue components and favors immune evasion by degrading opsonins. In *Staphylococcus aureus*, Plg binding is in part mediated by cell surface fibronectin-binding proteins (FnBPs), but the underlying molecular mechanism is not known. Here, we use single-cell and single-molecule techniques to demonstrate that FnBPs capture Plg by a sophisticated activation mechanism involving fibrinogen (Fg), another ligand found in the blood. We show that while FnBPs bind to Plg through weak (∼200-pN) molecular bonds, direct interaction of the adhesins with Fg through the high-affinity dock, lock, and latch mechanism dramatically increases the strength of the FnBP-Plg bond (up to ∼2,000 pN). Our results point to a new model in which the binding of Fg triggers major conformational changes in the FnBP protein, resulting in the buried Plg-binding domains being projected and exposed away from the cell surface, thereby promoting strong interactions with Plg. This study demonstrated a previously unidentified role for a ligand-binding interaction by a staphylococcal cell surface protein, i.e., changing the protein orientation to activate a cryptic biological function.

## IMPORTANCE

*Staphylococcus aureus* captures human plasminogen (Plg) via cell wall fibronectin-binding proteins (FnBPs), but the underlying molecular mechanism is not known. Here we show that the forces involved in the interaction between Plg and FnBPs on the *S. aureus* surface are weak. However, we discovered that binding of fibrinogen to FnBPs dramatically strengthens the FnBP-Plg bond, therefore revealing an unanticipated role for Fg in the capture of Plg by *S. aureus*. These experiments favor a model where Fg-induced conformational changes in FnBPs promote their interaction with Plg. This work uncovers a previously undescribed activation mechanism for a staphylococcal surface protein, whereby ligand-binding elicits a cryptic biological function.

## INTRODUCTION

The bacterial pathogen *Staphylococcus aureus* produces a variety of cell wall-anchored (CWA) proteins that play important roles in staphylococcal infections (1). Among these, the fibronectin-binding proteins (FnBPs) FnBPA and FnBPB (FnBPA/B) fulfil multiple functions (2). FnBPs mediate biofilm formation, including by clinically relevant resistant strains (3–5). Whereas the C-terminal domain binds to fibronectin, the N-terminal A domain binds to fibrinogen (Fg) by the high-affinity dock, lock, and latch (DLL) mechanism (6–8). DLL binding involves sequential conformational changes in subdomains N2 and N3 (N2N3) within the A region and results in the formation of highly stabilized complexes. FnBPs also play an important role in the accumulation phase of biofilm formation, by mediating low-affinity homophilic bonds between cells (4, 9). In addition, it has recently been shown that FnBPs are responsible for capturing host plasminogen (Plg) by *S. aureus* (10) and allow it to be activated to form plasmin. Plg capture has been demonstrated in a variety of invasive pathogens and is of medical importance as surface-bound plasmin enables bacteria to degrade the opsonins IgG and C3b, to dissolve fibrin clots, and to promote bacterial spread by cleaving tissue components (11–13).

Despite the great significance of Plg binding in staphylococcal pathogenesis, the molecular mechanism involved is still undefined. Two crucial and yet still unsolved issues are those of elucidating the nature of the molecular interactions driving the capture of Plg and determining whether FnBP-Fg and FnBP-Plg interactions interfere with each other. Here we address these issues using newly developed atomic force microscopy (AFM) modalities (14, 15). The results show that, while bacterial cells expressing FnBPs bind Plg through weak interactions, incubation of the cells with Fg dramatically increases the strength of the FnBP-Plg bond, thus highlighting an unexpected role for Fg in the capture of Plg by *S. aureus*. These experiments point to a model in which the DLL interaction between Fg and FnBPs triggers conformational changes in the adhesin, enabling the projection of the buried Plg-binding subdomains and their exposure beyond other cell surface components. The Fg-dependent capture mechanism of FnBPs is likely to be of significance for staphylococcal infections, by enhancing the ability of *S. aureus* to bind to and activate the nascent serum serine protease, which promotes destruction of opsonins, thus enhancing immune evasion and spread of bacteria in infected tissues. This activation mechanism could represent a potential target for the design of competitive inhibitors capable of blocking the spread of methicillin-resistant *S. aureus* in infected tissues.

## RESULTS

### FnBPA and FnBPB bind to the same kringle of Plg but do so through different subdomains.

**FIG 1:**
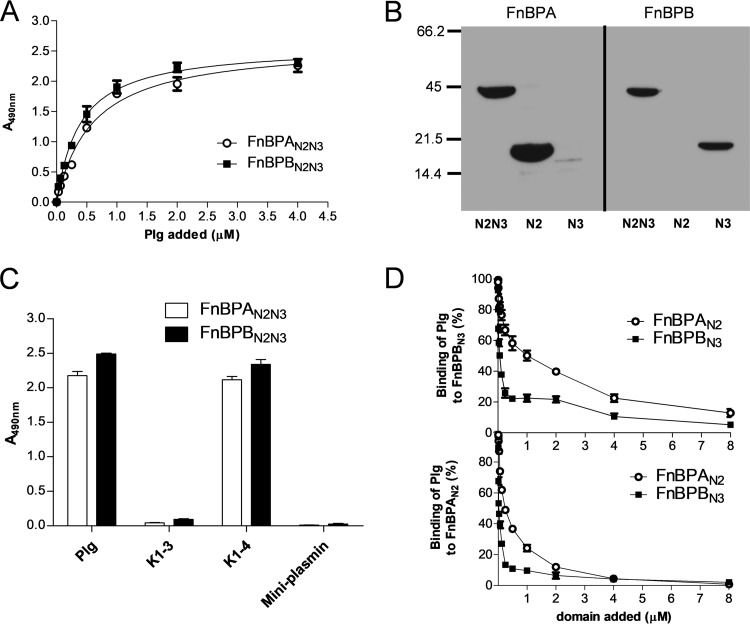
Recombinant FnBPA and FnBPB bind to plasminogen through different subdomains. (A) Recombinant FnBPA_N2N3_ and FnBPB_N2N3_ were immobilized on the surface of microtiter wells, and their binding was tested with increasing concentrations of Plg. Bound Plg was detected with rabbit antibodies to human Plg followed by HRP-conjugated goat anti-rabbit IgG. (B) Purified recombinant FnBPA_N2N3_ and FnBPB_N2N3_ and single FnBPA_N2_, FnBPA_N3_, FnBPB_N2_, and FnBPB_N3_ subdomains were subjected to SDS-PAGE, transferred to nitrocellulose membranes, and probed with human Plg followed by rabbit anti-Plg serum and then HRP-conjugated goat anti-rabbit IgG. (C) Microtiter plates coated with FnBPA_N2N3_ and FnBPB_N2N3_ were incubated with equimolar concentrations of Plg or Plg fragments K1-K3, K1-K4, and mini-Plg. Bound Plg and Plg fragments were detected as described for panel A. (D) Recombinant FnBPA_N2_ (lower panel) or FnBPB_N3_ (upper panel) was immobilized onto microtiter plates and incubated with Plg in the presence of increasing concentrations of soluble FnBPA_N2_ or FnBPB_N3_. Bound Plg was detected as described for panel A. Binding in the absence of potential inhibitor was set to 100%. The data points in panels A, C, and D represent means and errors of results of three independent experiments.

FnBPs have recently been shown to bind to Plg (10), and yet it is unclear exactly which adhesin subdomains are involved and which region of Plg contains the adhesin binding site. We tested the ability of increasing amounts of Plg to bind to purified recombinant N2N3 subdomains of FnBPA (FnBPA_N2N3_; amino acid residues 194 to 511) and FnBPB (FnBPB_N2N3_; amino acid residues 163 to 480) immobilized in microtiter wells (Fig. 1A). The dissociation constant (*K*_*d*_) values were calculated from the Plg saturation kinetics data and found to be 0.60 µM and 0.38 µM for FnBPA_N2N3_ and FnBPB_N2N3_, respectively. These values, in line with the 0.53 µM value measured for FnBPB_N2N3_ by surface plasmon resonance (SPR) analysis (10), indicate that the N2N3 subdomains of the two adhesins bind Plg with similar levels of affinity. We then studied Plg binding to either the N2 subdomain or the N3 subdomain of FnBPA (FnBPA_N2_) and FnBPB (FnBPB_N3_) using Western blot analysis. Figure 1B shows that FnBPA_N2_ and FnBPB_N3_ strongly bound to Plg, in contrast to FnBPA_N3_ and FnBPB_N2_, for which no binding was detected. This indicates that the two adhesins interact with Plg through different subdomains.

To determine which region of the Plg protein is involved in the interaction, we used an enzyme-linked immunosorbent assay (ELISA)-like experiment to evaluate the ability of FnBPs to bind to different Plg truncates comprising kringle 1 to kringle 3, kringle 1 to kringle 4, or mini-Plg (i.e., kringle 5 along with the C-terminal region of the protein). Figure 1C indicates that while kringle 1 to 3 and kringle 5 failed to bind to FnBPs, the combination of kringle 1 to kringle 4 bound to FnBPA_N2N3_ and FnBPB_N2N3_ in a fashion similar to that seen with the full-length Plg. This leads us to believe that kringle 4 is the sole binding domain for FnBPs, a belief which was further confirmed by showing that the interaction of immobilized FnBPA_N2_ or FnBPB_N3_ with Plg was competitively inhibited by both soluble FnBPA_N2_ and soluble FnBPB_N3_ (Fig. 1D). These observations reveal that FnBPA and FnBPB bind the same unique Plg kringle, with similar levels of affinity, and yet do so using different subdomains.

### Forces between *S. aureus* bacteria and plasminogen.

**FIG 2:**
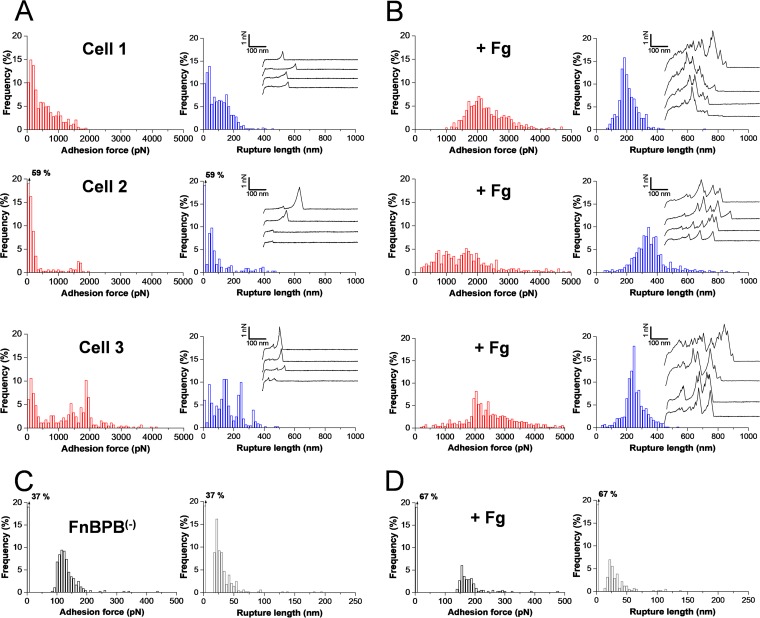
Single-cell force spectroscopy of the *S. aureus*-plasminogen interaction. (A and B) Adhesion force and rupture length histograms with representative retraction force profiles obtained by recording force-distance curves in PBS between different FnBPB^(+)^ cells and Plg substrates, in the absence (A) or presence (B) of 0.1 mg ⋅ ml^−1^ Fg. (C and D) Force data obtained under the same conditions for a FnBPB^(−)^ cell, in the absence (C) or presence (D) of 0.1 mg ⋅ ml^−1^ Fg. All curves were obtained using a contact time of 1 s, a maximum applied force of 250 pN, and approach and retraction speeds of 1,000 nm s^−1^.

**FIG 3:**
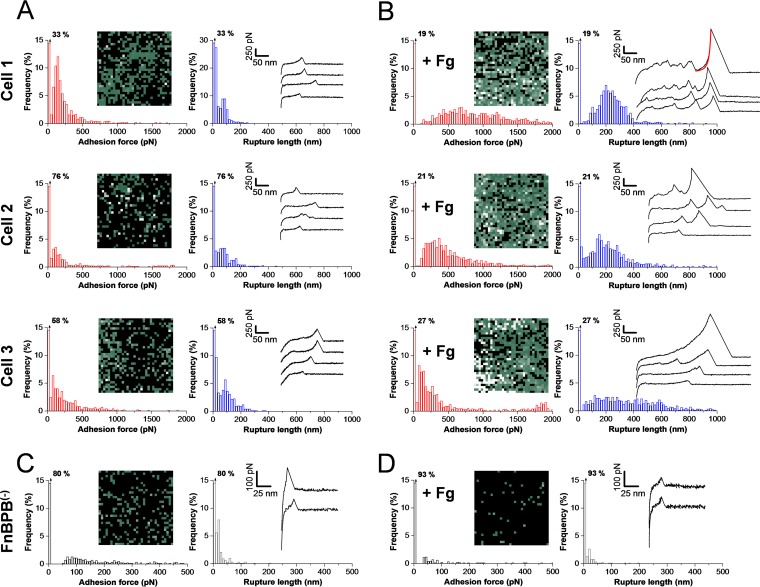
Single-molecule force spectroscopy demonstrated that fibrinogen dramatically increases the strength of the FnBPB-plasminogen bond. (A and B) Adhesion force maps (500 nm by 500 nm) and histograms, rupture length histograms, and representative retraction force profiles obtained by recording force-distance curves in PBS between Plg tips and different FnBPB^(+)^ cells, in the absence (A) or presence (B) of 0.1 mg ⋅ ml^−1^ Fg. (C and D) Force data obtained under the same conditions for a FnBPB^(−)^ cell, in the absence (C) or presence (D) of 0.1 mg ⋅ ml^−1^ Fg. All curves were obtained using a contact time of 250 ms, a maximum applied force of 250 pN, and approach and retraction speeds of 1,000 nm s^−1^.

To gain insight into the molecular interactions guiding the capture of Plg by cell surface-located FnBPs, we quantified the forces corresponding to interaction between *S. aureus* bacteria and Plg by means of single-cell force spectroscopy (SCFS) (16, 17) (Fig. 2). We analyzed cells displaying full-length FnBPs, expressed from a plasmid in *S. aureus* strain SH1000 defective in clumping factor A (ClfA) and ClfB, and in FnBPA and FnBPB [here, *S. aureus* FnBPA^(+)^ and FnBPB^(+)^ cells]. While both proteins were investigated, FnBPB was chosen for detailed analysis. Single cells were attached onto colloidal cantilevers coated with polydopamine, a bioinspired polydopamine wet adhesive, and force-distance curves between the cell probes and Plg substrates were then recorded. In Fig. 2A, we present the adhesion forces, rupture lengths, and typical adhesive force curves obtained for 3 different FnBPB^(+)^ cells (representative of 12 cells from 4 independent cultures). A substantial number of curves featured large adhesion force peaks, typically in the 500-to-2,000-pN range and with 50-to-400-nm rupture length (for cell 1, 556 ± 420 pN and 106 ± 71 nm [means ± standard deviations {SD}] from *n* = 519 adhesive force curves; for cell 2, 249 ± 226 pN together with a few curves at 1,628 ± 148 pN and 117 ± 110 nm, *n* = 139; for cell 3, 1,601 ± 517 pN and 163 ± 88 nm, *n* = 299). Variations in adhesion probability, adhesion forces, and rupture lengths that we attribute to cellular heterogeneity were observed. Adhesion was dramatically reduced with FnBPB^(−)^ cells (Fig. 2C [showing one cell that is representative of five cells]), with a mean adhesion force of 141 ± 65 pN (*n* = 856 adhesive force curves obtained for five cells from two independent cultures), indicating that they originated from specific FnBPB-Plg interactions. We note that some FnBPB^(+)^ cells (cells 2 and 3; see Fig. 2A) featured maximum adhesion forces at around 1,500 to 2,000 pN. While their physical origin is unclear, they are likely to reflect the simultaneous breakage of multiple FnBPB-Plg interactions. As our single-molecule data (see Fig. 3) show that the level of strength of single FnBPB-Plg bonds is ∼200 pN, this would imply that the 2,000-pN forces result from the rupture of 10 bonds loaded in parallel. Lastly, we found that FnBPA^(+)^ cells featured interaction forces that were quite similar to those seen with FnBPB^(+)^ cells (see Fig. S1A in the supplemental material).

10.1128/mBio.01067-17.1 FIG S1  Single-cell force spectroscopy shows that FnBPA mediates the binding of plasminogen by *S. aureus* just like FnBPB. (A and B) Adhesion force and rupture length histograms with representative retraction force profiles obtained by recording force-distance curves in PBS between different FnBPA^(+)^ cells and Plg substrates, in the absence (A) or presence (B) of 0.1 mg ⋅ ml^−1^ Fg. All curves were obtained using a contact time of 1 s, a maximum applied force of 250 pN, and approach and retraction speeds of 1,000 nm s^−1^. Download FIG S1, PDF file, 0.2 MB. Copyright © 2017 Herman-Bausier et al. 2017 Herman-Bausier et al. This content is distributed under the terms of the Creative Commons Attribution 4.0 International license.

Does FnBPA behave like FnBPB? In Fig. S2A, we show that FnBPA^(+)^ cells featured specific interactions similar to those detected on FnBPB^(+)^ cells (Fig. 3A). Consistent with the similar dissociation constants (Fig. 1A) and single-cell forces (Fig. S1), these results suggest that FnBPA and FnBPB bind Plg through the same type of molecular interaction. In addition, Fig. S2B reveals that the FnBPA-Plg bond, just like the FnBPB-Plg bond, was strengthened by the presence of Fg (Fig. 3B). This finding supports the idea that FnBPA and FnBPB capture Plg through similar mechanisms.

10.1128/mBio.01067-17.2 FIG S2  Single-molecule force spectroscopy reveals similar levels of binding strength and fibrinogen activation for FnBPA and FnBPB. (A and B) Adhesion force maps (500 nm by 500 nm) and histograms, rupture length histograms, and representative retraction force profiles obtained by recording force-distance curves in PBS between Plg tips and different FnBPA^(+)^ cells, in the absence (A) or presence (B) of 0.1 mg ⋅ ml^−1^ Fg. All curves were obtained using a contact time of 250 ms, a maximum applied force of 250 pN, and approach and retraction speeds of 1,000 nm s^−1^. Download FIG S2, PDF file, 0.2 MB. Copyright © 2017 Herman-Bausier et al. 2017 Herman-Bausier et al. This content is distributed under the terms of the Creative Commons Attribution 4.0 International license.

### Fibrinogen promotes the interaction between *S. aureus* and plasminogen.

As the N-terminal A domain of FnBPs binds Plg and Fg, both of which are important components of the blood, we then tested the hypothesis that Fg promotes the capture of Plg. Figure 2B shows that addition of Fg at 0.1 mg ml^−1^ dramatically changed the adhesive interactions between FnBPB^(+)^ cells and Plg, with the mean adhesion force and rupture length increasing from 775 ± 667 pN and 104 ± 94 nm to 2,104 ± 744 pN and 263 ± 91 nm (means ± SD from 3 cells representative of a total of 12 cells). Unlike the case with native cells, force profiles displayed complex shapes with multiple peaks, implying that multiple complex molecular bonds were formed. Interestingly, rupture distances were consistent with the length of fully unfolded FnBPBs. Indeed, assuming that each amino acid contributes 0.36 nm to the contour length of a polypeptide chain, the length of a fully extended adhesin (948 amino acids) is expected to be 341 nm, which, given the error values associated the measurements, is in the range of the observed extensions. The longer (up to 500-nm) distances sometimes observed may reflect the stretching of Plg molecules. The fact that shorter (∼100-nm) ruptures were observed in the absence of Fg supports the notion that full protein unfolding occurs only at high loading forces. Fg did not enhance the adhesion of FnBPB^(−)^ cells (Fig. 2D; one cell representative of five cells from two independent cultures), confirming that this effect results from the interaction between Fg and FnBPBs on the cell surface. Results obtained for FnBPA^(+)^ cells (Fig. S1B) were similar to those obtained for FnBPB^(+)^ cells, which suggests that the two adhesins, although they interact with different subdomains of FnBPs, bind Plg through similar Fg-dependent interactions.

### Fibrinogen increases the strength of the FnBP-plasminogen bond.

What is the mechanism by which Fg promotes bacterial adhesion? Does Fg directly activate Plg binding, or does it play an indirect role? To answer these questions, single-molecule force spectroscopy (SMFS) (16, 18) performed with Plg-terminated tips was used to measure the strength of single FnBP-Plg bonds on living bacteria (Fig. 3). Figure 3A shows adhesion force maps, maximum adhesion forces, rupture lengths, and force profiles recorded from 3 FnBPB^(+)^ cells (similar data were obtained from a total of 12 cells from 5 independent cultures). Single adhesion peaks were detected, with rather weak forces of 188 ± 107 pN magnitude (means ± SD; data represent 1,159 adhesive curves obtained from 3 cells representative of 12 cells from 4 independent cultures). These forces originated from FnBPB-Plg bonds as they were strongly reduced [drop of adhesion frequency from 40% to 21%; *n* = 10,240 curves on 10 FnBPB^(+)^ cells and 5,120 curves on 5 FnBPB^(−)^ cells] using FnBPB^(−)^ cells (Fig. 3C; one cell representative of five cells). We believe that mostly single bonds were detected because Plg molecules were attached to the AFM tip at low density (19), and single well-defined adhesion peaks with sharp ruptures were always observed. The ∼200-pN force is in the range of the strength of FnBP homophilic bonds and much lower than the force of a DLL interaction (e.g., ∼2 nN for the SdrG-Fg bond [16]), thus confirming earlier data showing that the FnBPB-Plg bond does not involve a DLL mechanism (10). The observed rupture lengths, 76 ± 58 nm (1,351 curves; 3 cells), are much shorter than the length of fully extended adhesins, implying that the bond ruptured before complete protein unfolding. We also note that adhesion maps revealed that FnBP proteins were exposed at rather high density on the cell surface and yet showed variations from 1 cell to another. In contrast with the other staphylococcal adhesins SdrG and Cna (16, 20), FnBPB proteins were randomly distributed without evidence of clustering.

Remarkably, the strength of the FnBP-Plg interaction was strongly enhanced in the presence of Fg (Fig. 3B), with a clear increase in the binding probability (for cell 1, an increase from 67% to 81%; for cell 2, an increase from 24% to 79%; for cell 3, an increase from 42% to 73%) and in the binding force (for cell 1, an increase from 175 ± 82 pN to 898 ± 403 pN; for cell 2, an increase from 154 ± 83 pN to 619 ± 355 pN; for cell 3, an increase from 176 ± 98 pN to 561 ± 586 pN). The rupture length also increased to 258 ± 134 nm (means ± SD; three cells), which is very close to the value range determined for whole cells (Fig. 2B) and suggests, again, that FnBPB molecules were fully unfolded at high loading forces. Stronger adhesion forces may originate from either of two possible mechanisms; i.e., direct interaction of Fg with FnBPBs on the cell surface promoting further binding to Plg or adsorption of Fg to the Plg tip favoring its interaction with the cell surface. Therefore, two control experiments were performed in order to establish whether Fg is capable of binding to Plg. First, an ELISA-type experiment was carried out where soluble Fg was tested for binding to immobilized Plg. No significant binding of Fg to Plg was observed even at the highest concentration of the ligand used (Fig. S3A). Second, the forces between Plg tips and Fg-coated substrates were measured and found to be very weak (Fig. S3B), thus demonstrating that Fg hardly binds to Plg at all.

10.1128/mBio.01067-17.3 FIG S3  Fibrinogen does not bind to plasminogen. (A) Plg was immobilized on microtiter wells and tested for binding to Fg. Bound Fg was detected with anti-Fg mouse IgG followed by HRP-conjugated rabbit anti-mouse IgG. Data shown are from three independent experiments. (B) Adhesion force histogram and force profiles obtained in PBS between a Plg tip and an Fg substrate. Similar data were obtained in triplicate experiments. Download FIG S3, PDF file, 0.1 MB. Copyright © 2017 Herman-Bausier et al. 2017 Herman-Bausier et al. This content is distributed under the terms of the Creative Commons Attribution 4.0 International license.

One may argue that adhesins other than FnBPA/B and ClfA/B that are capable of binding to Fg might be present on the surface of SH1000 *S. aureus*. We therefore used SMFS with Fg tips to confirm that FnBPBs represent the major Fg-binding proteins on the cell surface (Fig. S4). FnBPB^(+)^ cells featured forces that were remarkably strong (1,968 ± 46 pN, 1,691 ± 42 pN, and 1,963 ± 45 pN for cells 1 to 3) (Fig. S4A) and in the range of force values measured for single high-affinity DLL interactions between SdrG and Fg (16). In contrast, adhesion forces were abolished on FnBPB^(−)^ cells (Fig. S4B), thus showing that the strong forces originated exclusively from FnBPB-Fg interactions. Taken together, these observations lead us to conclude that strengthening of the FnBPB-Plg interaction originates from the direct interaction of Fg with FnBPs. We propose that the DLL interaction between Fg and FnBPB triggers a major conformational change in the protein and therefore promotes further interaction with Plg.

10.1128/mBio.01067-17.4 FIG S4  Fibrinogen primarily binds to FnBPs on the cell surface. (A) Adhesion force maps (500 nm by 500 nm) and histograms, rupture length histograms, and representative retraction force profiles obtained by recording force-distance curves in PBS between Fg tips and 3 different FnBPB^(+)^ cells (representative of 8 different cells). (B) Force data obtained under the same conditions for a FnBPB^(−)^ cell (representative of 8 different cells). All curves were obtained using a contact time of 100 ms, a maximum applied force of 250 pN, and approach and retraction speeds of 1,000 nm s^−1^. Download FIG S4, PDF file, 0.3 MB. Copyright © 2017 Herman-Bausier et al. 2017 Herman-Bausier et al. This content is distributed under the terms of the Creative Commons Attribution 4.0 International license.

### Plasminogen binding by FnBP fragments is not influenced by fibrinogen.

**FIG 4:**
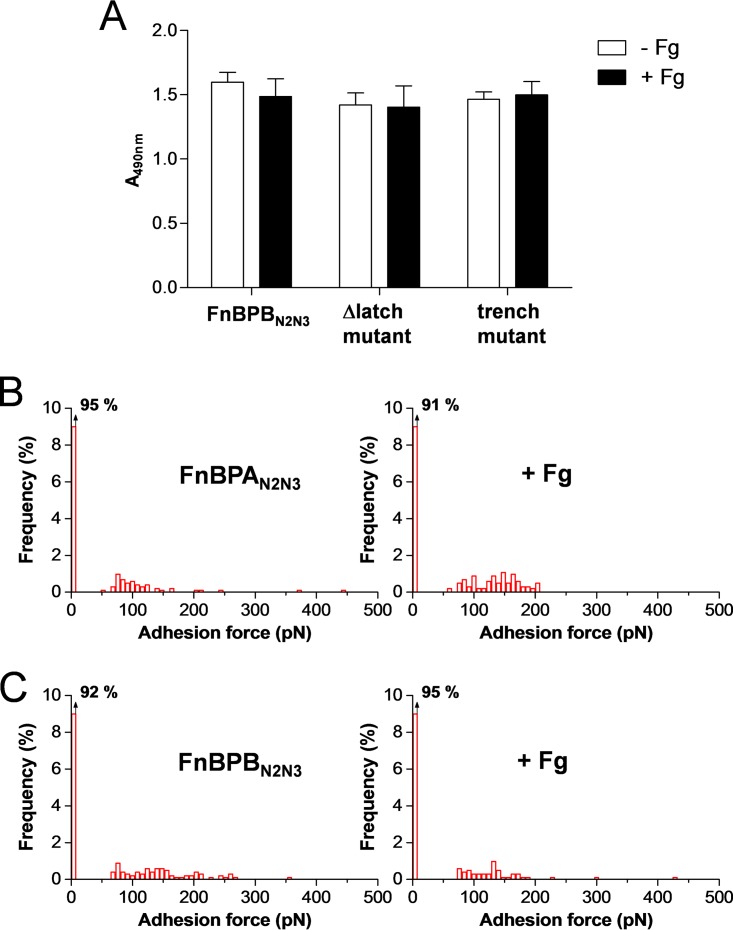
Binding of plasminogen to recombinant FnBPs is not influenced by fibrinogen. (A) Binding of Plg to FnBPB_N2N3_ and the derivative FnBPB_N2N3_ latch and FnPBB_N2N3_ trench mutants immobilized on microtiter wells, in the presence of saturating amounts (2 mM) of Fg. Bound Plg was detected with rabbit antibodies followed by HRP-conjugated goat anti-rabbit IgG. The data shown are the means ± SD of results of three independent experiments. (B and C) Single-molecule adhesion force data obtained in PBS between Plg tips and either FnBPA_N2N3_ (B) or FnBPB_N2N3_ (C) immobilized on a substrate, in the absence (left) or presence (right) of 0.1 mg ⋅ ml^−1^ Fg. Similar data were obtained in duplicate experiments.

Finally, we investigated the effect of Fg on the binding of Plg by recombinant subdomains of the ligand-binding A-region of FnBPs. First, we designed an ELISA-type experiment where Plg was allowed to bind to immobilized FnBPB_N2N3_ in the presence of Fg (Fig. 4A). No significant enhancing activity of Fg was observed with full FnBPB_N2N3_ fragments. Further, both the C-terminal truncate (FnBPB; region 163–463) and the trench mutant (FnBPB; region 163–480 [N321A/F314A]), which lack the ability to interact with Fg, showed very similar Plg-binding profiles in the presence of Fg, confirming that the Fg and Plg binding sites on the FnBPB_N2N3_ domains do not overlap. We also used SMFS to probe the interaction forces between Plg tips and recombinant FnBP_N2N3_ subdomains immobilized on a solid surface (Fig. 4B and C). Force curves obtained for FnBPA_N2N3_ and FnBPA_N2N3_ documented very low adhesion probability and adhesion forces and were not altered by Fg. These results are very different from the forces measured on live cells, leading us to believe that FnBP fragments attached to a solid substrate do not allow freedom for the protein to undergo conformational changes when Fg is added. This finding highlights the notion that Fg-induced conformational changes can occur only in the cellular context and not on purified immobilized proteins, thus emphasizing the importance of studying the mechanism driving the capture of Plg directly in live cells.

## DISCUSSION

*S. aureus* is a leading cause of hospital-acquired infections. Staphylococcal infections caused by strains like methicillin-resistant *S. aureus* (MRSA) that are resistant to multiple antibiotics are particularly difficult to eradicate. Like other invasive pathogens, *S. aureus* can capture Plg from human plasma (10, 21–25), enabling it to form plasmin. This process is of medical significance as plasmin is a serine protease that degrades many blood plasma proteins and cleaves tissue components, thereby promoting bacterial spread in infected tissues. Recently, FnBPs were found to be major Plg-binding proteins on the *S. aureus* cell surface (10). FnBPB binds Plg and Fg simultaneously, implying the presence of distinct nonoverlapping binding sites. Despite the fact that both ligands are components of the blood plasma, it is not known whether they interfere with each other during the capture of Plg by *S. aureus* or whether such dual binding activity is of biological significance.

We have measured the molecular forces driving the capture of Plg by FnBPs and have shown the key role of Fg in activating this interaction. Unlike traditional assays, live-cell nanoscopy experiments enable us to study the binding mechanisms of adhesins in their cellular context and thus in their biologically relevant conformations and orientations. Our main findings can be summarized as follows. First, FnBPA and FnBPB bind to kringle 4 of Plg through their N2 and N3 subdomains, respectively. Second, the strength of single FnBP-Plg bonds is ∼200 pN, which is much lower than the strength of the DLL interaction between staphylococcal surface adhesins and Fg. Third, binding of Fg to FnBPs on the cell surface dramatically strengthens FnBP-Plg bonds, with forces of up to ∼2,000 pN, thus providing direct evidence that Fg strongly favors Plg interactions. Control experiments demonstrated that this activation process originates from the direct binding of Fg to FnBPs on the cell surface. In the presence of these high forces, the adhesin is fully unfolded; whether this contributes to increase the capture of Plg remains to be clarified. Fg has no effect on recombinant FnBPBs, highlighting the need to probe the adhesins in their native cellular environment.

**FIG 5:**
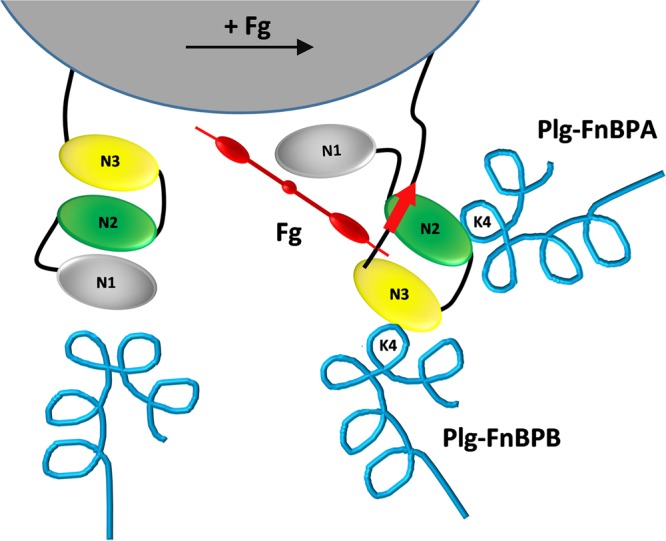
Molecular mechanism driving the capture of Plg by *S. aureus*. In the absence of Fg (left), the FnBPN2N3 subdomains that contain the Plg binding sites are buried and hidden by other cell surface components, thus hampering their strong interaction with surrounding Plg molecules (in blue). Binding of Fg (in red) by the dock, lock, and latch (DDL) mechanism induces sequential conformational changes in the N2N3 subdomains that lead to their exposure on the cell surface and favor strong direct interaction with kringle 4 of Plg molecules.

Collectively, our results support the notion of a new activation mechanism for the capture of Plg by *S. aureus*. As illustrated in Fig. 5, the DLL interaction of Fg with FnBPs on the cell surface triggers a major conformational change in the adhesins, resulting in the buried Plg binding sites in the N2 and N3 subdomains being projected and exposed for optimal Plg binding. Thus, this report unveils an unanticipated role for the DLL interaction, that is, modulating the orientation of a staphylococcal adhesin to enhance its adhesive function. To the best of our knowledge, this is the first time that high-affinity ligand binding by a staphylococcal cell surface protein was found to elicit a cryptic biological function. It is tempting to speculate that other staphylococcal CWA proteins may have evolved similar mechanisms to activate their multiple functions.

The ability of *S. aureus* to capture Plg from serum or plasma and to facilitate its activation to the potent serine protease plasmin—by the endogenously expressed plasminogen activator staphylokinase or host plasminogen activators—is likely to be of benefit to the pathogen via several scenarios during infection. First, plasmin can degrade host opsonin IgG and complement C3b, thus protecting bacteria from neutrophil-mediated phagocytosis and killing (13). Second, active plasmin bound to the bacterial cell surface promotes spreading in host tissue by degradation of the host extracellular matrix (26). Third, biofilm formation by *S. aureus* in the presence of plasma is strongly influenced by the presence of staphylokinase (27). Thus, activated plasmin degrades the fibrin and Fg in the matrix that forms between cells in the *in vivo* biofilm. This implies that captured Plg/plasmin promotes dissemination during biomaterial-related infection.

The Fg-dependent interactions of FnBPs could represent a potential target for antibacterial therapy. The design of antibodies or peptides capable of blocking both Fg and Plg binding sites on FnBPs could be used to inhibit the capture and the subsequent formation of plasmin by *S. aureus*, thus blocking bacterial spread in host tissues. Such an approach could be particularly useful to treat soft tissue infections by strains that are resistant to multiple antibiotics.

## MATERIALS AND METHODS

### Bacterial strains and growth conditions.

We used *S. aureus* strain FnBP^(−)^ (SH1000 *clfA clfB fnbA fnbB*), which is defective in clumping factors A and B and fibronectin binding proteins A and B (9). FnBP^(−)^ cells were grown overnight in Trypticase soy broth (TSB), washed once with TSB, subcultured into TSB at a 1:100 dilution, and allowed to grow to an optical density at 600 nm (OD_600_) of 0.4. *S. aureus* strains FnBPA^(+)^ and FnBPB^(+)^ are derivatives of strain SH1000 *clfA clfB fnbA fnbB* carrying plasmid pFNBPA4 or pFNBPB4 expressing fibronectin binding protein A or B from strain 8325-4, respectively (28). For expression of FnBP^(+)^, cells were grown overnight in TSB with chloramphenicol (10 µg ⋅ ml^−1^), washed once in TSB, subcultured into TSB at a 1:100 dilution, and allowed to grow to an OD_600_ of 0.4 in TSB plus chloramphenicol.

### Plasmid and DNA manipulation.

Plasmid DNA (see Table S1 in the supplemental material) was isolated using a Wizard Plus SV miniprep kit (Promega, Madison, WI) according to the manufacturer’s instructions and transformed into *Escherichia coli* TOPP3 cells using standard procedures (29). Transformants were screened by restriction analysis and verified by DNA sequencing (Eurofins Genomics, Milan, Italy). Chromosomal DNA was extracted using a bacterial genomic DNA purification kit (Edge Biosystems, Gaithersburg, MD). Primers listed in Table S2 were purchased from Integrated DNA Technologies, Inc. (Leuven, Belgium), and used to amplify the sequence for cloning into pQE30. Restriction digestions and ligations were carried out using enzymes from New England Biolabs (Ipswich, MA) according to the manufacturer’s protocols. DNA purification was carried out using a Wizard SV gel and PCR cleanup system (Promega).

10.1128/mBio.01067-17.5 TABLE S1  Plasmids. Download TABLE S1, PDF file, 0.1 MB. Copyright © 2017 Herman-Bausier et al. 2017 Herman-Bausier et al. This content is distributed under the terms of the Creative Commons Attribution 4.0 International license.

10.1128/mBio.01067-17.6 TABLE S2  Primers (F is forward, and R is reverse). Download TABLE S2, PDF file, 0.1 MB. Copyright © 2017 Herman-Bausier et al. 2017 Herman-Bausier et al. This content is distributed under the terms of the Creative Commons Attribution 4.0 International license.

### Expression and purification of recombinant proteins.

Recombinant proteins FnBPB (163–480, FnBPB_N2N3_), FnBPB (163–308, FnBPB_N2_), FnBPB (309–480, FnBPB_N3_), FnBPA (194–511, FnBPA_N2-N3_), FnBPA (194–336) (FnBPA_N2_), FnBPA (337–511, FnBPA_N3_), and FnBPB (163–463) latch truncated were expressed from pQE30 (Qiagen, Chatsworth, CA) in *E. coli* TOPP3 (Stratagene). Overnight starter cultures were diluted 1:50 in Luria broth containing ampicillin (100 μg/ml) and incubated with shaking until the culture reached an optical density at 600 nm (A_600_) of 0.4 to 0.6. Recombinant protein expression was induced by addition of isopropyl 1-thio-β-d-galactopyranoside (0.5 mM) and continued for 2 h. Bacterial cells were harvested by centrifugation, frozen at −80°C, and purified from cell lysates by Ni^+2^ affinity chromatography using a HiTrap chelating column (GE Healthcare). Recombinant FnBPB (163–480) N312A/F314A trench mutant was expressed with a His_6_ N-terminal affinity tag using *E. coli* vector and purified on a HiTrap chelating column. The purity of the recombinant proteins was assessed to be 98% by SDS-PAGE, Coomassie brilliant blue staining, and densitometry analysis. A bicinchoninic acid protein assay (Pierce) was used to measure concentrations of purified proteins.

### Plasminogen and fibrinogen.

Plg was purified from plasma by affinity chromatography on a Lys-Sepharose column (30). Human Fg (Calbiochem) was purified on a gelatin-Sepharose column to remove contaminating fibronectin. The purity of the proteins was assessed by 10% SDS-PAGE and Coomassie brilliant blue staining. Kringle 1 to 3 and kringle 1 to 4 were purchased from Sigma and MyBiosource (San Diego), respectively. The mini-Plg (residues Val^442^ to Asn^790^) was obtained by digestion of Plg with porcine pancreatic elastase (Sigma), as described previously (31, 32).

### Antibodies.

Polyclonal antisera against purified human Plg or Fg were raised in rabbit or mouse by routine immunization procedures using Plg or Fg as the antigen. Goat anti-rabbit or rabbit anti-mouse horseradish peroxidase (HRP)-conjugated secondary antibodies were purchased from DakoCytomation (Glostrup, Denmark).

### ELISA-based binding experiment.

The ability of surface-coated staphylococcal proteins to interact with soluble Plg was determined using an ELISA-based solid-phase binding assay. Microtiter wells were coated overnight at 4°C with a mixture of 200 ng/well of each bacterial protein dissolved in 0.1 M sodium carbonate (pH 9.5). The plates were washed with 0.5% (vol/vol) Tween 20–phosphate-buffered saline (PBST). To block additional protein-binding sites, the wells were treated for 1 h at 22°C with bovine serum albumin (BSA) (2% [vol/vol])–phosphate-buffered saline (PBS). The plates were then incubated for 1 h with the appropriate amounts of Plg. After several washings with PBST, 100 μl of anti-Plg rabbit polyclonal IgG diluted 1:2,500 was added to the wells and the reaction mixtures were incubated for 90 min. The plates were washed and then incubated for 1 h with HRP-conjugated goat anti-rabbit IgG diluted 1:1,000. After washing, *o*-phenilenediamine dihydrochloride was added, and the absorbance at 490 nm was determined using an ELISA plate reader (Bio-Rad, Richmond, CA). To analyze binding of Fg to immobilized Plg, microtiter plate wells were coated with 200 ng Plg, blocked with BSA, and incubated with increasing amounts of human Fg. Bound fibrinogen was detected by addition of a mouse anti-Fg serum diluted 1:2,500 followed by HRP-conjugated rabbit anti-mouse IgG (1:1,000) as reported above. To identify Plg kringles involved in FnBPA_N2N3_ and FnBPB_N2N3_ binding, microtiter plates were coated with 0.5 μg/well of FnBP proteins and incubated with Plg, Plg fragments K1 to K3 or K1 to K4, or mini-Plg (1 μM). Protein bound to FnBPA or FnBPB was detected by the use of rabbit anti-Plg serum diluted 1:2,500 followed by HRP-conjugated goat anti-rabbit IgG (1:1,000).

### Western blotting.

Recombinant staphylococcal proteins were boiled for 3 min in sample buffer (0.125 M Tris-HCl, 4% [wt/vol] SDS, 20% [vol/vol] glycerol, 10% [vol/vol] β-mercaptoethanol, 0.002% [wt/vol] bromophenol blue) and separated by 12.5% (wt/vol) PAGE. Proteins were electroblotted onto a nitrocellulose membrane (GE Healthcare), and the membrane was blocked overnight at 4°C with 5% (wt/vol) skim milk (Sigma)–PBS. The membrane was incubated with 1 μg/ml Plg–PBST for 1 h at 22°C, washed, and further incubated with rabbit polyclonal antibody against Plg (1:5,000) for 1 h at 22°C. Following several washings with PBST, the membrane was incubated for 1 h with HRP-conjugated goat anti-rabbit IgG (1:10,000). Finally, the blot was developed using an ECL Advance Western blotting detection kit (GE Healthcare). An ImageQuant LAS 4000 mini-biomolecular imager (GE Healthcare) was used to capture images of the bands.

### Plasminogen and FnBP-coated substrates.

Plg and FnBP fragments were attached to gold-coated surfaces via *N*-hydroxysuccinimide (NHS) surface chemistry. To this end, gold-coated glass substrates were immersed overnight in ethanol solutions containing 1 mM 16-mercaptohexadecanoic acid (Sigma) and 11-mercapto-1-undecanol (Sigma) at a molar ratio of 1:90 and were then rinsed with ethanol. Substrates were immersed for 30 min in a solution containing 10 mg ml^−1^ NHS (Sigma) and 25 mg ml^−1^ 1-ethyl-3-(3-dimethylaminopropyl)-carbodiimide (EDC) (Sigma), rinsed, and then incubated with 0.2 mg ml^−1^ Plg or 0.2 mg ml^−1^ FnBP fragments mixed with PBS for 1 h, followed by rinsing with PBS.

### Single-cell force spectroscopy.

Bacterial cell probes were obtained as previously described (17, 33). Briefly, single silica microspheres (Bangs Laboratories) (6.1-μm diameter) were attached with a thin layer of UV-curable glue (NOA 63; Norland Edmund Optics) on triangular tipless cantilevers (NP-O10; Microlevers, Bruker Corporation) by the use of a Nanoscope VIII multimode AFM (Bruker Corporation, Santa Barbara, CA). The cantilever was then immersed for 1 h in 10 mM Tris buffer–150 mM NaCl solution (pH 8.5) containing 4 mg ml^−1^ dopamine hydrochloride (Sigma) (99%). The probe was then rinsed in Tris buffer–150 mM NaCl solution (pH 8.5) and used directly for cell probe preparation. The nominal spring constant of the colloidal probe cantilever was ∼0.06 N m^−1^ as determined by the thermal noise method.

For cell probe preparation, 50 μl of a suspension of ca. 1 × 10^6^ cells was transferred into a glass petri dish containing Plg-coated substrates mixed with PBS. The colloidal probe was brought into contact with a bacterium. Single bacteria were attached on the center of the colloidal probes using a Bioscope Catalyst AFM (Bruker, Santa Barbara, CA) equipped with a Zeiss Axio observer Z1 stand and a Hamamatsu camera (model C10600). The cell probe was then positioned over the Plg substrates without dewetting. Forces corresponding to single-cell interactions with Plg substrates were measured at room temperature (20°C) by recording multiple force curves on five different spots. For activation experiments, Fg from human plasma (Sigma F3879) was added into the AFM chamber to reach a final concentration of 0.1 mg ml^−1^. Adhesion and rupture length histograms were generated by considering, for every force curve, the maximum adhesion force and the rupture length of the last peak, respectively.

### Single-molecule force spectroscopy on live cells.

SMFS analysis was performed with live cells at room temperature (20°C) in PBS buffer using a Nanoscope VIII multimode AFM (Bruker Corporation, Santa Barbara, CA) and oxide-sharpened microfabricated Si_3_Ni_4_ cantilevers (Microlevers; Bruker Corporation) with a nominal spring constant of ∼0.01 N m^−1^. The spring constants of the cantilevers were measured using the thermal noise method. Bacterial cells were immobilized by mechanical trapping into porous polycarbonate membranes (Millipore, Billerica, MA) with a pore size similar to the cell size (34). After filtration of a cell suspension was performed, the filter was gently rinsed with PBS, carefully cut into sections (1 cm by 1 cm), and attached to a steel sample puck using a small piece of double-face adhesive tape and the mounted sample was transferred into the AFM liquid cell while avoiding dewetting. Plg functionalized tips were obtained using polyethylene glycol (PEG)-benzaldehyde linkers (19). Prior to functionalization, cantilevers were washed with chloroform and ethanol, placed in a UV-ozone cleaner for 10 min, immersed in an ethanolamine solution (5 g ethanolamine–10 ml dimethyl sulfoxide [DMSO]) and maintained overnight, and then washed three times with DMSO and two times with ethanol and dried with N2. The ethanolamine-coated cantilevers were immersed for 2 h in a solution prepared by mixing 1 mg acetal-PEG-NHS dissolved in 0.5 ml of chloroform with 10 μl trimethylamine and were then washed with chloroform and dried with N2. Cantilevers were further immersed for 5 min in a 1% citric acid solution, washed in ultrapure water (ELGA LabWater), and then covered with a 200-μl droplet of PBS solution containing Plg (2 μM) to which 2 μl of a 1 M NaCNBH_3_ solution had been added. After 50 min, cantilevers were incubated with 5 μl of a 1 M ethanolamine solution in order to passivate unreacted aldehyde groups and then washed with and stored in buffer. For activating experiments, we added Fg from human plasma (Sigma F3879) into the AFM chamber to reach a final concentration of 0.1 mg ml^−1^. Adhesion and rupture length histograms were generated by considering, for every force curve, the adhesion force of the last peak and its rupture length.

### Single-molecule force spectroscopy on model surfaces.

SMFS measurements were performed for assays of Plg tips and substrates functionalized with FnBP fragments (prepared as described above) using a Nanoscope VIII multimode AFM (Bruker Corporation, Santa Barbara, CA). In some experiments, Fg (Sigma F3879) was added into the AFM chamber to reach a final concentration of 0.1 mg ml^−1^. Adhesion and rupture length histograms were generated by considering, for every force curve, the adhesion force of the last peak and its rupture length.
