# Supplementary material for: Fibrinogen Activates the Capture of Human Plasminogen by Staphylococcal Fibronectin-Binding Proteins
Source: mBio. 2017 Sep 5;8(5):e01067-17. doi: 10.1128/mBio.01067-17 (PMC5587908; doi:10.1128/mBio.01067-17)
Supplement: FIG S1 [file mbo004173467sf1.pdf]

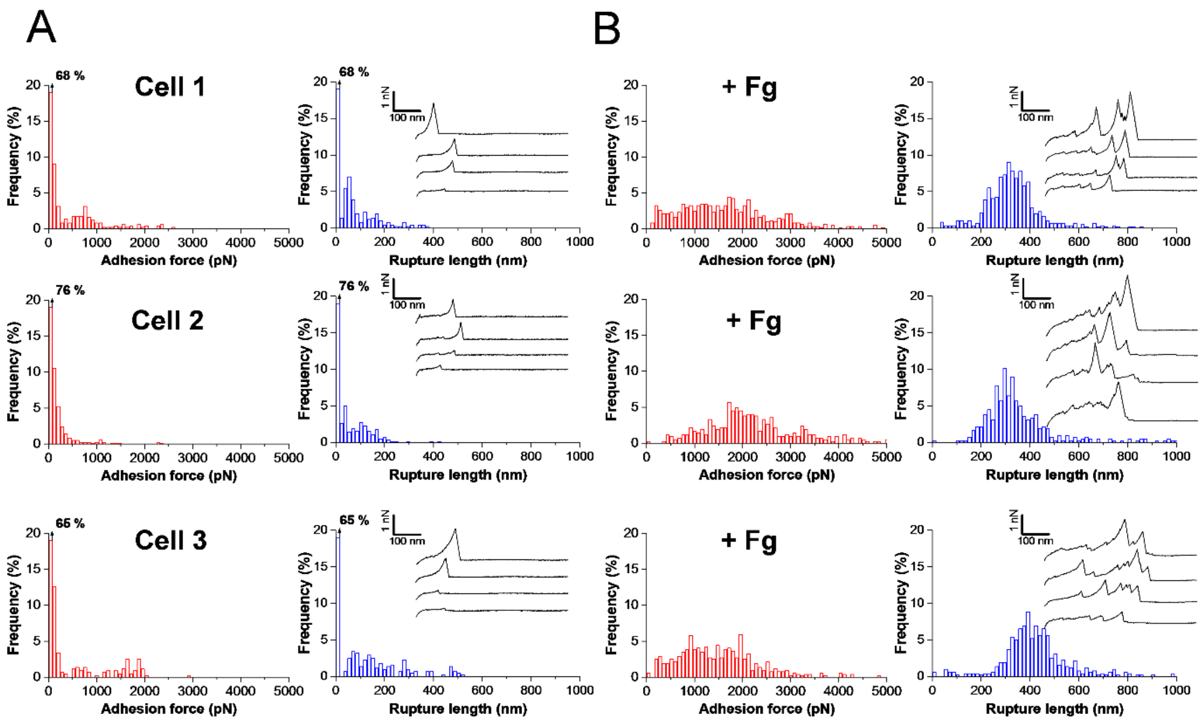

Fig. S1. Single-cell force spectroscopy shows that FnBPA mediates the binding of plasminogen by *S. aureus* just like FnBPB. (*A, B*) Adhesion force and rupture length histograms with representative retraction force profiles obtained by recording force-distance curves in PBS between different FnBPA$^{(+)}$ cells and Plg-substrates, in the absence (*A*) or presence (*B*) of 0.1 mg.ml^{-1} Fg. All curves were obtained using a contact time of 1 s, a maximum applied force of 250 pN, and approach and retraction speeds of 1,000 nm s^{-1}.
