# Supplementary material for: Fibrinogen Activates the Capture of Human Plasminogen by Staphylococcal Fibronectin-Binding Proteins
Source: mBio. 2017 Sep 5;8(5):e01067-17. doi: 10.1128/mBio.01067-17 (PMC5587908; doi:10.1128/mBio.01067-17)
Supplement: FIG S3 [file mbo004173467sf3.pdf]

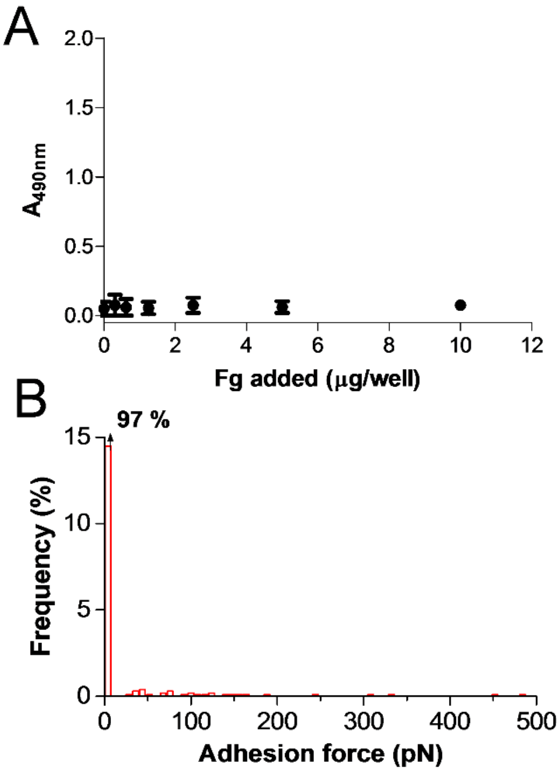

Fig. S3. Fibrinogen does not bind to plasminogen. (*A*) Plg was immobilized on microtiter wells and tested for binding to Fg. Bound Fg was detected with anti-Fg mouse IgG followed by HRP-conjugated rabbit anti-mouse IgG. Data from three independent experiments are shown. (*B*) Adhesion force histogram and force profiles obtained in PBS between a Plg-tip and a Fg-substrate. Similar data were obtained in triplicate experiments.
