# Supplementary material for: Fibrinogen Activates the Capture of Human Plasminogen by Staphylococcal Fibronectin-Binding Proteins
Source: mBio. 2017 Sep 5;8(5):e01067-17. doi: 10.1128/mBio.01067-17 (PMC5587908; doi:10.1128/mBio.01067-17)
Supplement: FIG S4 [file mbo004173467sf4.pdf]

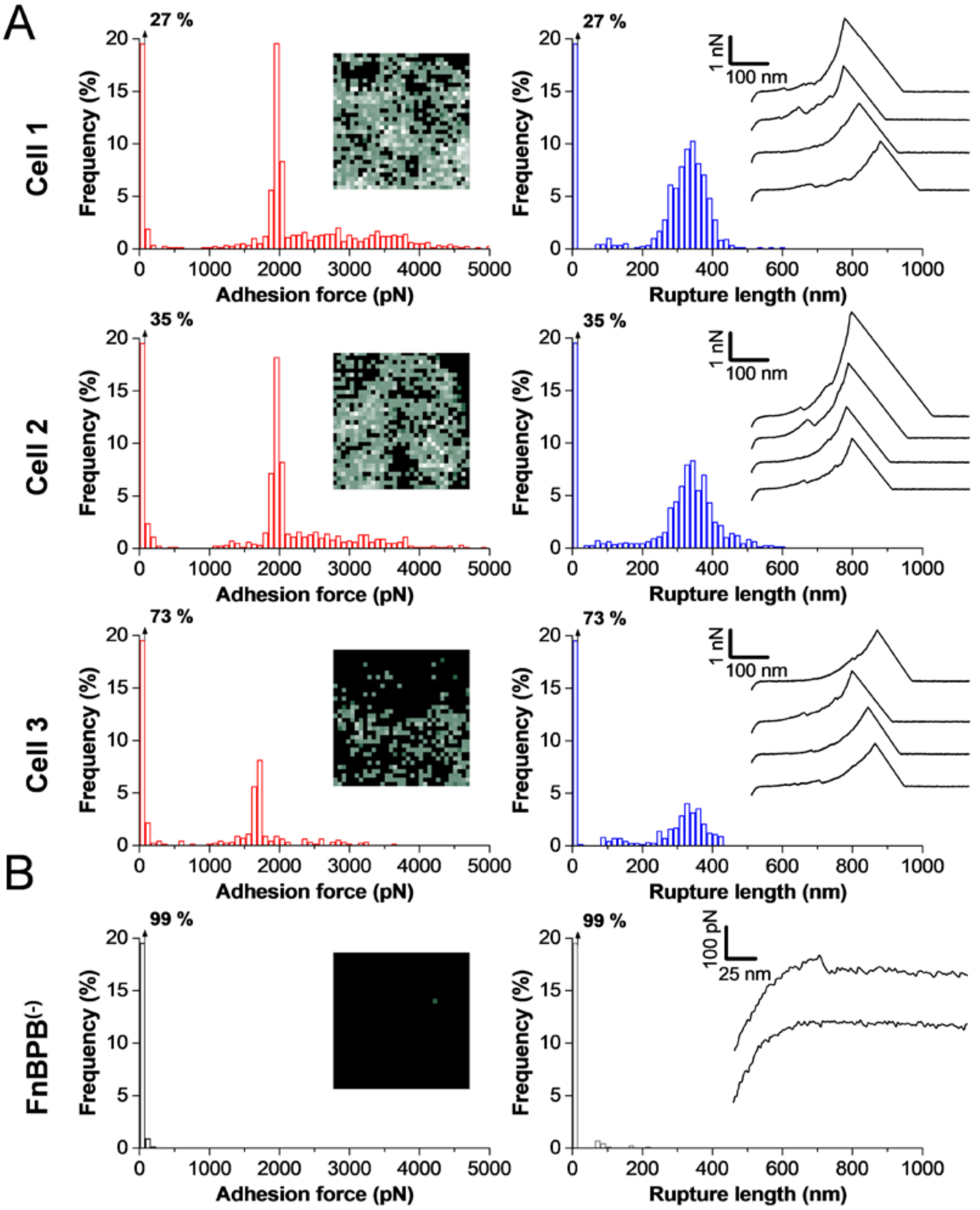

Fig. S4. Fibrinogen primarily binds to FnBPs on the cell surface. (*A*) Adhesion force maps and histograms, rupture length histograms and representative retraction force profiles obtained by recording force-distance curves in PBS between Fg-tips and 3 different FnBPB$^{(+)}$ cells (representative out of 8 different cells). (*B*) Force data obtained in the same conditions for a FnBPB$^{(-)}$ cell (representative out of 8 different cells). All curves were obtained using a contact time of 100 ms, a maximum applied force of 250 pN, and approach and retraction speeds of 1,000 nm s^{-1}.
