# Supplementary material for: Fibrinogen Activates the Capture of Human Plasminogen by Staphylococcal Fibronectin-Binding Proteins
Source: mBio. 2017 Sep 5;8(5):e01067-17. doi: 10.1128/mBio.01067-17 (PMC5587908; doi:10.1128/mBio.01067-17)
Supplement: TABLE S1 [file mbo004173467st1.pdf]

Table S1. Plasmids.

Plasmid	Features	Marker	Source/Reference
pQE30	*E. coli* vector for the expression of hexa-His-tagged recombinant proteins	Amp^R	Qiagen
pQE30::rFnBPB(163-480)	pQE30 derivative encoding the N2N3 subdomain of FnBPB from S. aureus 8325–4	Amp^R	FEBS J. (2011) 278, 2359–2371
pQE30::rFnBPB(163-463)	pQE30 derivative encoding residues 163–463 of FnBPB from S. aureus 8325–4	Amp^R	FEBS J. (2011) 278, 2359–2371
pQE30::rFnBPB(163-480) N312A/F314A	pQE30 derivative encoding the N2N3 subdomain of FnBPB from S. aureus 8325-4 with mutations encoding the changes N312A and F314A	Amp^R	FEBS J. (2011) 278, 2359–2371
pQE30::rFnBPB(163-308)	pQE30 derivative encoding the N2 subdomain of FnBPB from S. aureus 8325–4	Amp^R	JBC (2016) 291, 18148-18162
pQE30::rFnBPB(309-480)	pQE30 derivative encoding the N3 subdomain of FnBPB from S. aureus 8325–4	Amp^R	JBC (2016) 291, 18148-18162
pQE30::rFnBPA(194-511)	pQE30 derivative encoding the N2N3 subdomain of FnBPA from S. aureus 8325–4	Amp^R	This study
pQE30::rFnBPA(194-336)	pQE30 derivative encoding the N2 subdomain of FnBPB from S. aureus 8325–4	Amp^R	This study
pQE30::rFnBPA(337-511)	pQE30 derivative encoding the N3 subdomain of FnBPB from S. aureus 8325–4	Amp^R	This study

Amp^R: ampicillin resistance
