# Supplementary material for: Fibrinogen Activates the Capture of Human Plasminogen by Staphylococcal Fibronectin-Binding Proteins
Source: mBio. 2017 Sep 5;8(5):e01067-17. doi: 10.1128/mBio.01067-17 (PMC5587908; doi:10.1128/mBio.01067-17)
Supplement: TABLE S2 [file mbo004173467st2.pdf]

Table S2. Primers (F is forward, and R is reverse).

Primer	Sequence (5'–3')[a]	5'-Restriction site
rFnBPA(194–511) F	CGCGGATCCGGCACAGATGTAACAAGTAAAG	BamHI
rFnBPA(194–511) R	GACGTCGACTTAATTTTTCTCATTTCCGTTCTC	SalI
rFnBPA(194-336) F	CGCGGATCCGGCACAGATGTAACAAGTAAAG	BamHI
rFnBPA(194-336) R	GACGCGTCGACTTATTTAACATCTAATTCCTTTG	SalI
rFnBPA(337-511) F	CGCGGATCCTATAAAGATGGTATTGGG	BamHI
rFnBPA(337-511) R	GACGTCGACTTAATTTTTCTCATTTCCGTTCTC	SalI

[a]Restriction sites used for cloning are underlined.
